# Supplementary material for: BTLA contributes to acute-on-chronic liver failure infection and mortality through CD4+ T-cell exhaustion
Source: Nat Commun. 2024 Feb 28;15:1835. doi: 10.1038/s41467-024-46047-8 (PMC10901893; doi:10.1038/s41467-024-46047-8)
Supplement: Supplementary file 1 — Supplementary information [file 41467_2024_46047_MOESM1_ESM.pdf]

Supplementary Information

BTLA contributes to acute-on-chronic liver failure infection and mortality through $CD4^+$ T-cell exhaustion

Xueping Yu [1, 2, †, *], Feifei Yang [1, †], Zhongliang Shen [1, †], Yao Zhang [1, †], Jian Sun [1], Chao Qiu [1], Yijuan Zheng [2], Weidong Zhao [3], Songhua Yuan [4], Dawu Zeng [5], Shenyan Zhang [1], Jianfei Long [6], Mengqi Zhu [1], Xueyun Zhang [1], Jingwen Wu [1], Zhenxuan Ma [1], Haoxiang Zhu [1], Milong Su [2], Jianqing Xu [4], Bin Li [7], Richeng Mao [1*], Zhijun Su [2*], Jiming Zhang [1, 8, 9*]

[†] These authors contributed equally: Xueping Yu, Feifei Yang, Zhongliang Shen, and Yao Zhang.

*Correspondent emails: (J.Z.) jmzhang@fudan.edu.cn; (X.Y.) xpyu15@fudan.edu.cn; (Z.S.) su2366@sina.com; (R.M.) njxiaomao@163.com.

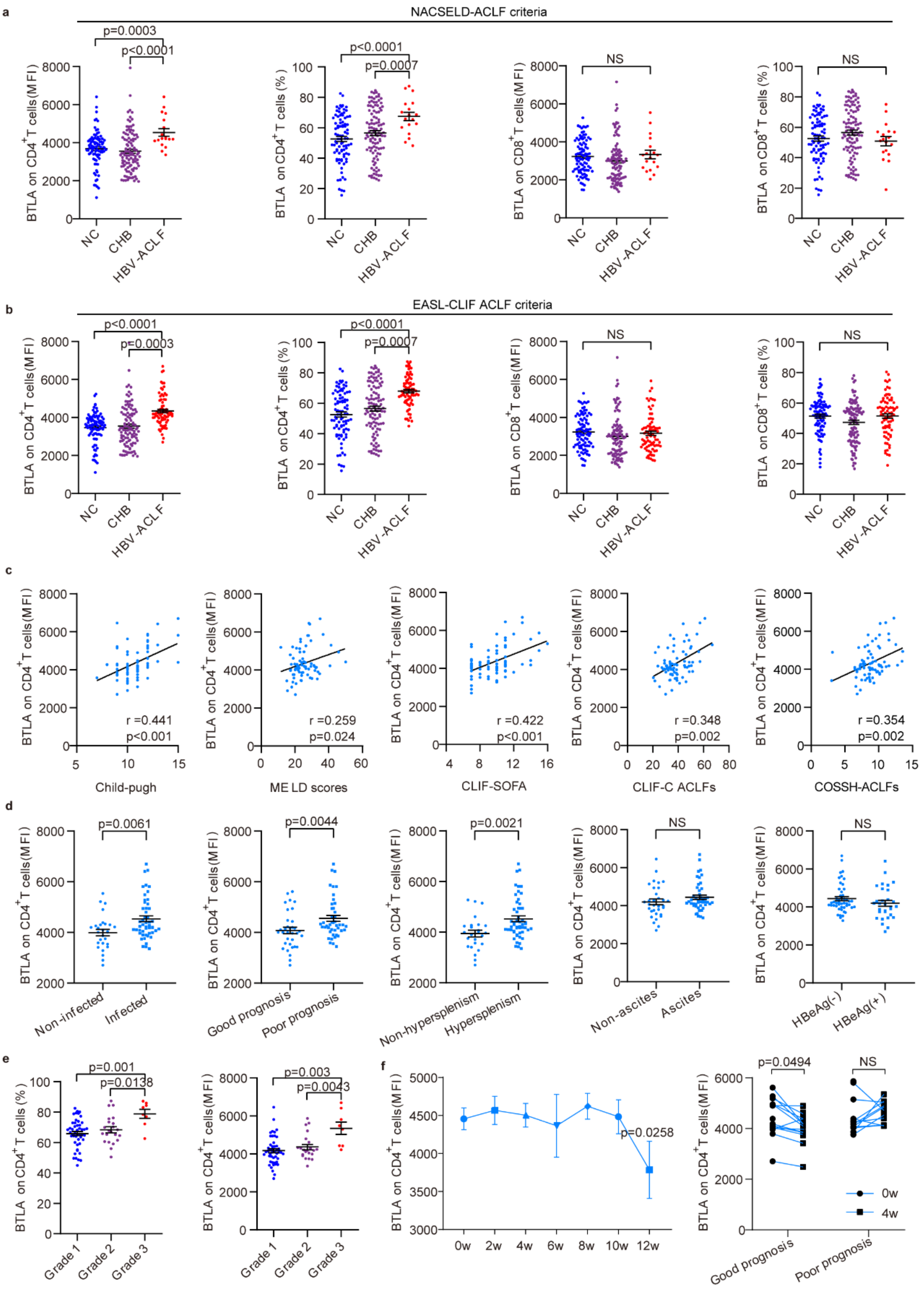

Supplementary Fig. 1 BTLA expression was significantly increased on circulation CD4^{+} T cells in HBV-ACLF patients and was positively correlated with prognosis and infectious complications. (a, b) Patients with HBV-ACLF who met the diagnostic criteria of NACSELD (a, NC: *n* = 90 donors, CHB: *n* = 104 donors, HBV-ACLF: *n* = 18 donors) or EASL-CLIF (b, NC: *n* = 90 donors, CHB: *n* = 104 donors, HBV-ACLF: *n* = 78 donors) had significantly increased expression of

BTLA on peripheral blood $CD4^+$ T cells compared to NC and CHB patients, while there was no significant difference in the BTLA expression of $CD8^+$ T cells. (c) The MFI of $CD4^+BTLA^+$ T cells was positively correlated with the severity of the disease (Child-pugh, MELD scores, CLIF-SOFA, CLIF-C ACLFs, and COSSH-ACLFs) in patients with HBV-ACLF who met the diagnostic criteria of EASL-CLIF (n = 78 donors). (d) Relationship between the MFI of $CD4^+BTLA^+$ T cells and complications (non-infected vs. infected: n = 28 vs. n = 50 donors; non-hypersplenism vs. hypersplenism: n = 25 vs. n = 53 donors; non-ascites vs. ascites: n = 32 vs. n = 46 donors), prognosis (good prognosis vs. poor prognosis: n = 34 vs. n = 44 donors), and HBeAg status (positive vs. negative: n = 48 vs. n = 30 donors) in patients with HBV-ACLF who met the diagnostic criteria of EASL-CLIF. (e) Changes in the MFI of $CD4^+BTLA^+$ T cells in the progression of HBV-ACLF who met the diagnostic criteria of EASL-CLIF (n = 78 donors). (f) The patients with a good prognosis of HBV-ACLF (n = 14 donors) had a significantly decreased MFI of $CD4^+BTLA^+$ T cells after 4 weeks compared with that before treatment. Data were calculated as mean ± SEM (a, b, d, e, f), Kruskal-Wallis H test followed by Dunn's multiple comparison test (a, b, e), Mann–Whitney U test (d, f), and Spearman tests (c). A two-sided P value $< .05$ was considered significant.

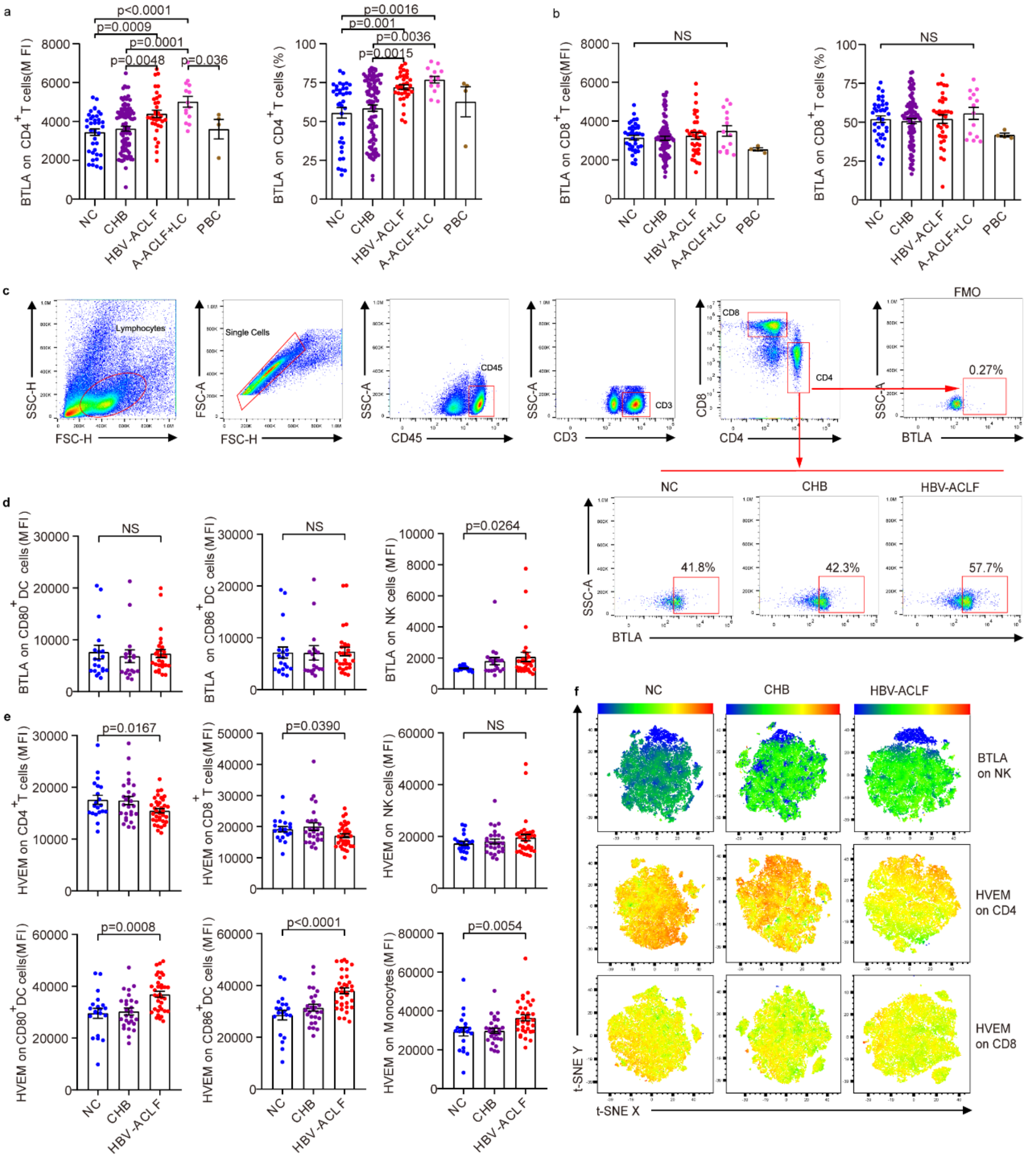

Supplementary Fig. 2 Expression of BTLA in ACLF patients of various etiologies, as well as BTLA and HVEM expression on T cells, NK cells, and dendritic cells (DC). (a, b) Expression of BTLA on CD4/CD8^{+} T cells in NC (n = 38 donors), CHB (n = 93 donors), HBV-ACLF (n = 35 donors), alcohol-induced ACLF and cirrhosis (n = 14 donors), and primary biliary cholangitis (PBC) patients (n = 4 donors). (c) Flow cytometry diagram of BTLA expression on intrahepatic CD4^{+} T cells in NC, CHB, and HBV-ACLF patients. (d) MFI of BTLA expression on NK cells but not on CD80/86^{+} DC increased in HBV-ACLF patients (n = 35 donors) compared with that in CHB patients (n = 27 donors) and NC (n = 20 donors). (e) MFI of HVEM expression on CD4/CD8^{+} T cells but not on NK cells decreased in patients with HBV-ACLF (n = 35 donors) compared with that in NC (n = 20 donors); HVEM levels on CD80/D86^{+} DC and monocytes were increased in patients with HBV-ACLF compared with those in NC and CHB patients (n = 27 donors). (f) T-distributed

stochastic neighbor embedding (t-SNE) was used to determine the expression of BTLA and HVEM on NK cells or CD4/CD8^{+} T cells in NC, CHB, and HBV-ACLF patients. Data were calculated as mean ± SEM (a, b, d, e), Kruskal-Wallis H test followed by Dunn's multiple comparison test (a, b, d, e). A two-sided *P* value < .05 was considered significant.

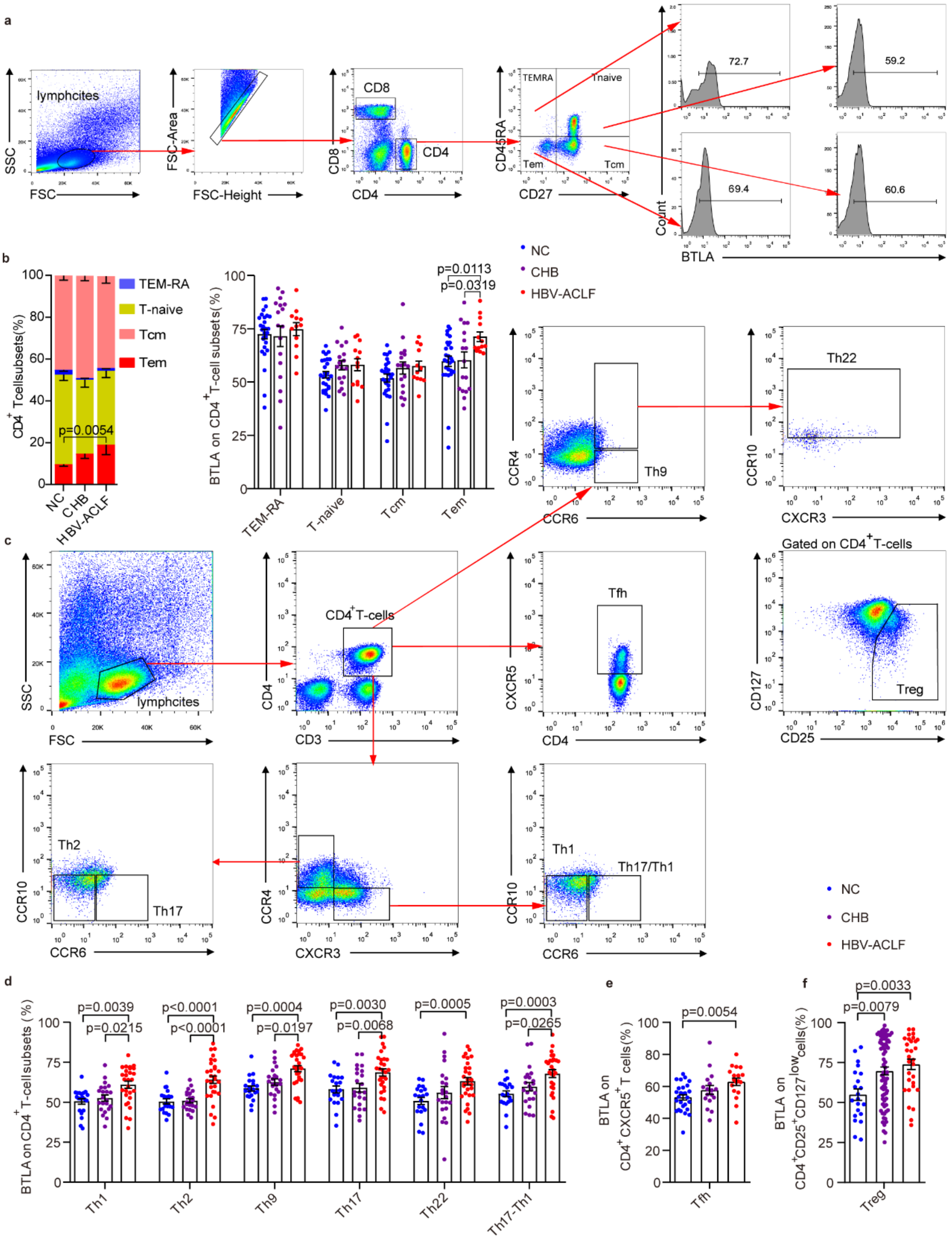

Supplementary Fig. 3 BTLA expression significantly increased on the Tem subtype, on all subgroups of circulation CD4^{+} T cells, and on intrahepatic CD4^{+} T cells in HBV-ACLF patients. (a) Flow cytometry diagram of BTLA expression on T-effector memory re-expressing CD45RA (TEM-RA), naïve T cells (T naïve),

central memory T cells (Tcm), and effector memory T cells (Tem, classified according to CD27 and CD45RA). (b) Frequencies of subtypes of $CD4^+$ T cells (TEM-RA, T naïve, Tcm, and Tem) and the expression of BTLA on these cell subtypes from NC (*n* = 27 donors), CHB (*n* = 22 donors), and HBV-ACLF patients (*n* = 13 donors). (c) Sequential gating strategy for the subgroups of $CD4^+$ T cells (Th1, Th2, Th9, Th17, Th17-Th1, Th22, Tfh, and Treg cells) [1]. (d) BTLA expression on the Th1, Th2, Th9, Th17, Th22, and Th17-Th1 cell subgroups increased in HBV-ACLF patients (*n* = 29 donors) compared with those in NC (*n* = 20 donors) and CHB patients (*n* = 22 donors). The same trend was observed in Tfh (e, NC: *n* = 27 donors, CHB: *n* = 17 donors, HBV-ACLF: *n* = 18 donors), and Treg subgroups (f, NC: *n* = 21 donors, CHB: *n* = 72 donors, HBV-ACLF: *n* = 30 donors). Data were calculated as mean ± SEM (b, d, e, f), Kruskal-Wallis H test followed by Dunn's multiple comparison test (b, d, e, f). A two-sided P value < .05 was considered significant.

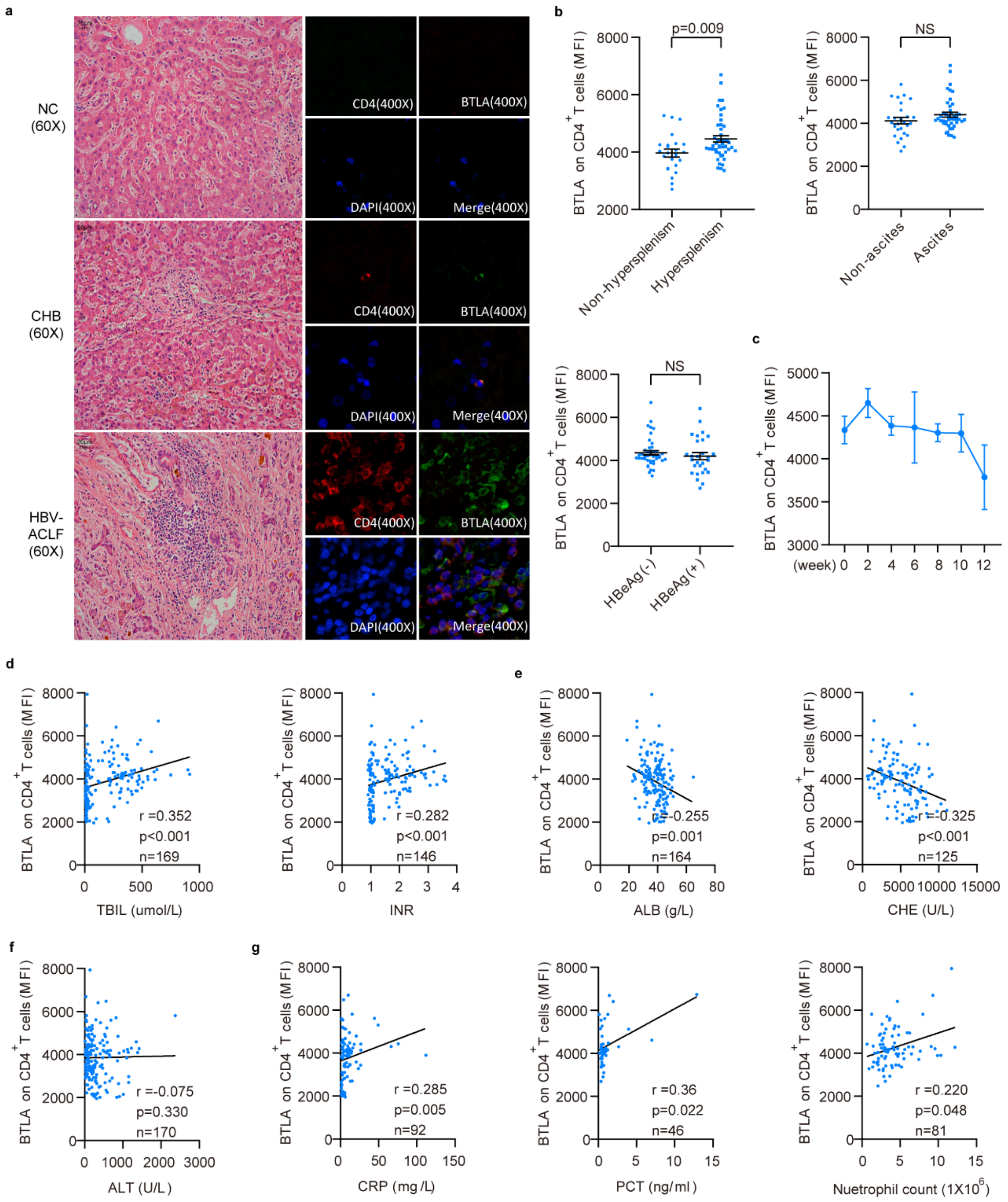

Supplementary Fig. 4 BTLA$^+$CD4$^+$ T cells were positively correlated with severity of disease, prognosis, and infectious complications. (a) Number of CD4$^+$BTLA$^+$ cells in liver tissue were significantly higher in HBV-ACLF patients than in NC or CHB patients. (b) There were no significant differences between HBV-ACLF patients with or without ascites complications (n = 27 vs. n = 44 donors) or between HBeAg-positive and HBeAg-negative patients (n = 43 vs. n = 28 donors). (c) After comprehensive treatment, the MFI of BTLA expression on CD4$^+$ T cells in patients with HBV-ACLF gradually decreased (n = 20 donors). (d-g) The frequency of CD4$^+$BTLA$^+$ T cells was positively correlated with physiological and biochemical indices of liver injury (total bilirubin (TBil), international normalized ratio (INR)) and systemic inflammation (neutrophil count, C-reactive protein (CRP), and procalcitonin (PCT)), but negatively correlated with compensatory indices of liver function

(Albumin (ALB) and cholinesterase (CHE)). Data were calculated as mean ± SEM (b, c), Mann–Whitney *U* test (b, c) and Spearman tests (d-g). A two-sided *P* value < .05 was considered significant.

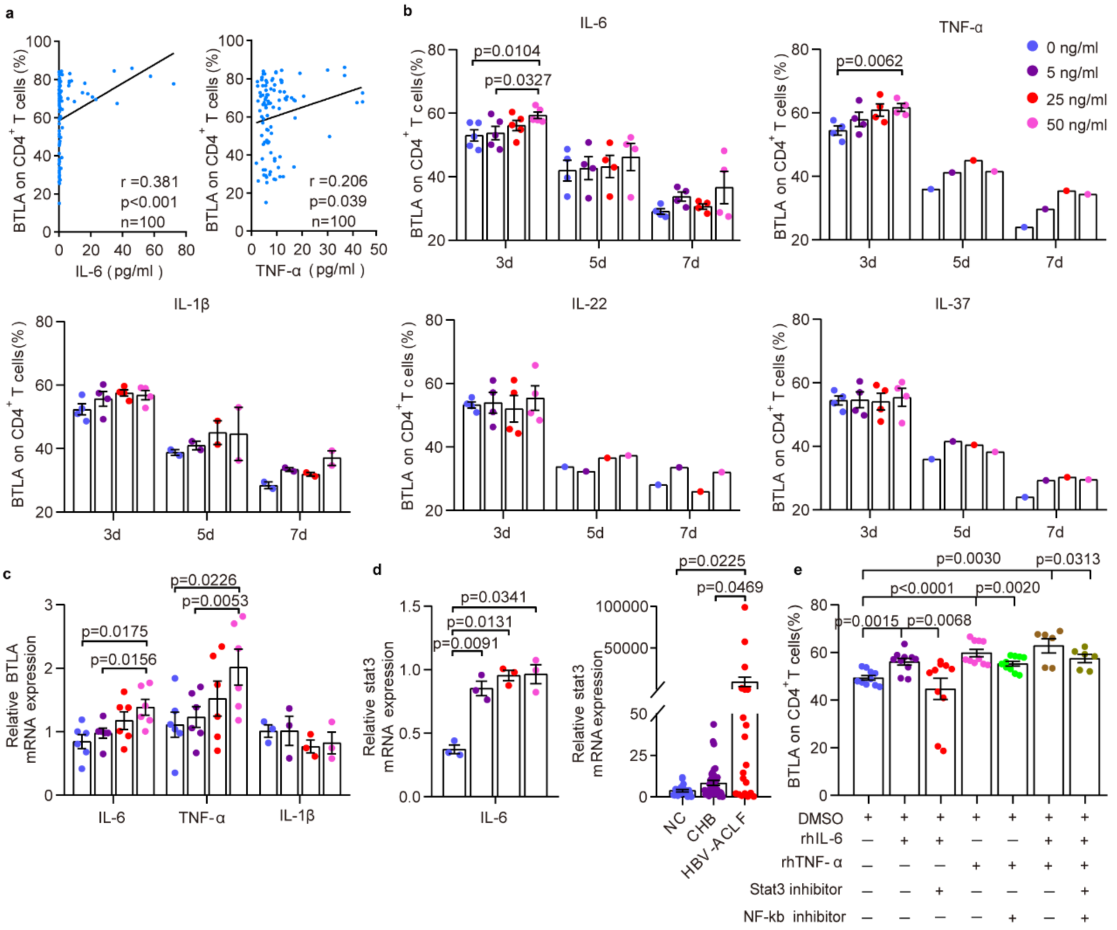

Supplementary Fig. 5 BTLA expression on CD4$^+$ T cells was induced by interleukin (IL)-6 and tumor necrosis factor (TNF)-α. (a) Levels of IL-6 (left) and TNF-α (right) were significantly and positively correlated with BTLA expression on CD4$^+$ T cells. (b) Expression of BTLA on CD4$^+$ T cells was higher at day 3 than at days 5 or 7 upon exposure of peripheral blood mononuclear cells (PBMC) to recombinant human (rh) IL-1β (*n* = 4 donors), rhIL-6 (*n* = 5 donors), rhIL-22 (*n* = 4 donors), rhIL-37 (*n* = 4 donors), and rhTNF-α (*n* = 4 donors). (c) rhIL-6 and rhTNF-α induced the up-regulation of *BTLA* mRNA levels in a dose-dependent manner (all *n* = 6 donors). (d) Levels of *Stat3* mRNA (right) in HBV-ACLF patients (*n* = 20 donors) were significantly higher than those in NC (*n* = 21 donors) and CHB patients (*n* = 39 donors), and rhIL-6 (left) significantly increased the levels of *Stat3* mRNA in a dose-dependent manner (*n* = 3 donors). (e) Exposure to rhIL-6 plus anti-stat3 or rhTNF-α plus anti-NF-κb resulted in a significant decrease in BTLA expression on CD4$^+$ T cells compared with exposure to only rhIL-6 or rhTNF-α (*n* = 10 donors). Data were calculated as mean ± SEM (b, c, d, e), Spearman tests (a), One-way ANOVA followed by Tukey's multiple comparison test (b, c, e), and Wilcoxon test (d). A two-sided *P* value < .05 was considered significant.

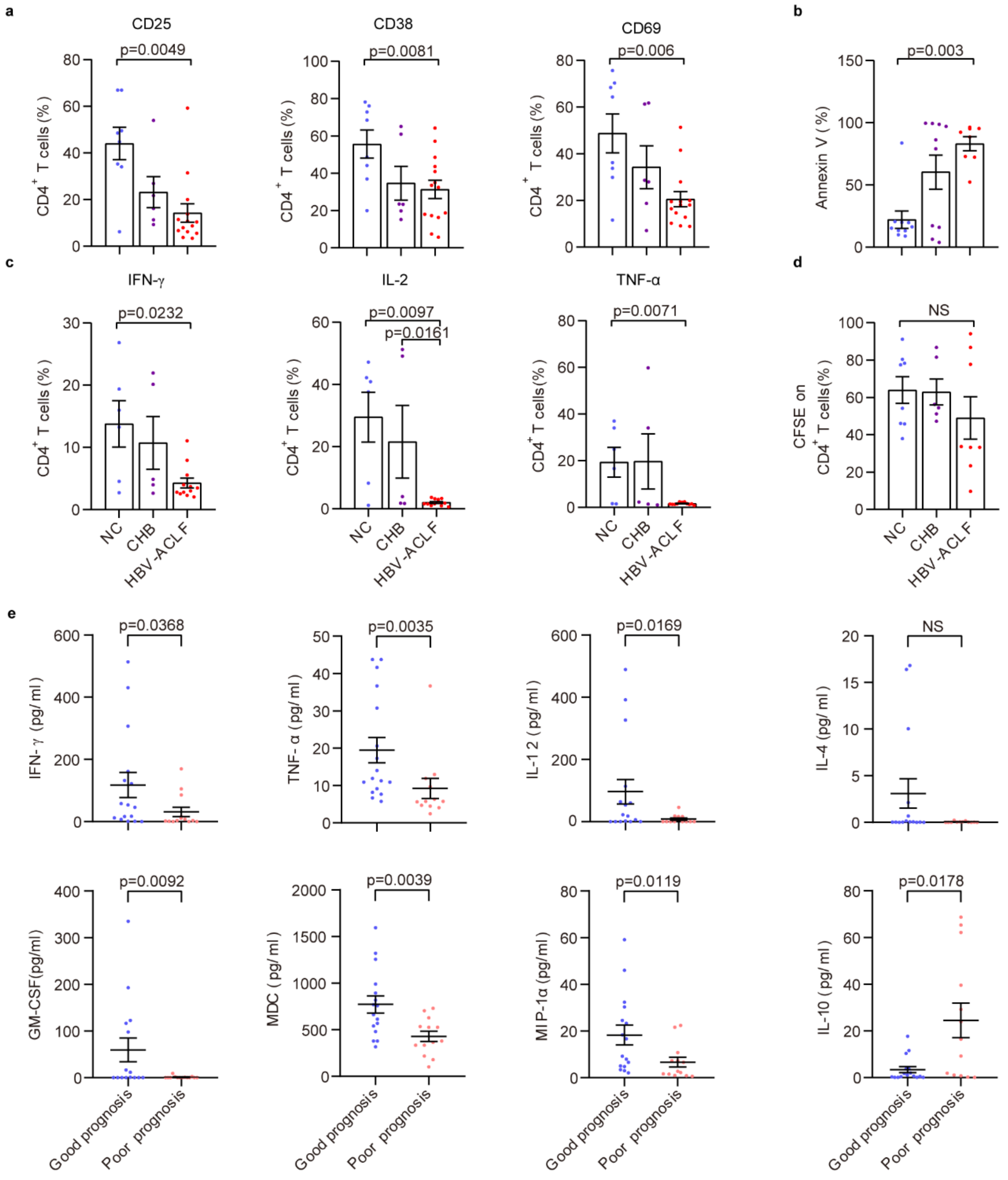

Supplementary Fig. 6 CD4$^+$ T cells tend to be exhausted in HBV-ACLF, and this exhaustion is associated with poor prognosis. (a, b, c, d) HBV-ACLF patients displayed a decreased ability for activation (NC: *n* = 8 donors, CHB: *n* = 6 donors, HBV-ACLF: *n* = 14 donors), proliferation (NC: *n* = 8 donors, CHB: *n* = 6 donors, HBV-ACLF: *n* = 8 donors), and secretory cytokines (NC: *n* = 6 donors, CHB: *n* = 5 donors, HBV-ACLF: *n* = 12 donors), but had an increased apoptosis rate (NC: *n* = 10 donors, CHB: *n* = 10 donors, HBV-ACLF: *n* = 8 donors) in CD4$^+$ T cells. (e) HBV-ACLF patients with poorer prognoses (*n* = 14 donors) displayed lower plasma levels of Th1-like (IFN-γ and TNF-α) and Th2-like (IL-12 and IL-4) cytokines, as well as other chemokines (GM-CSF, MDC, and MIP-1α), but higher levels of IL-10 than HBV-ACLF patients with better prognoses (*n* = 16 donors). Data were

calculated as mean ± SEM (a, b, c, d, e), Kruskal-Wallis H test followed by Dunn's multiple comparison test (a, b, c, d) and Mann–Whitney *U* test (e). A two-sided *P* value < .05 was considered significant.

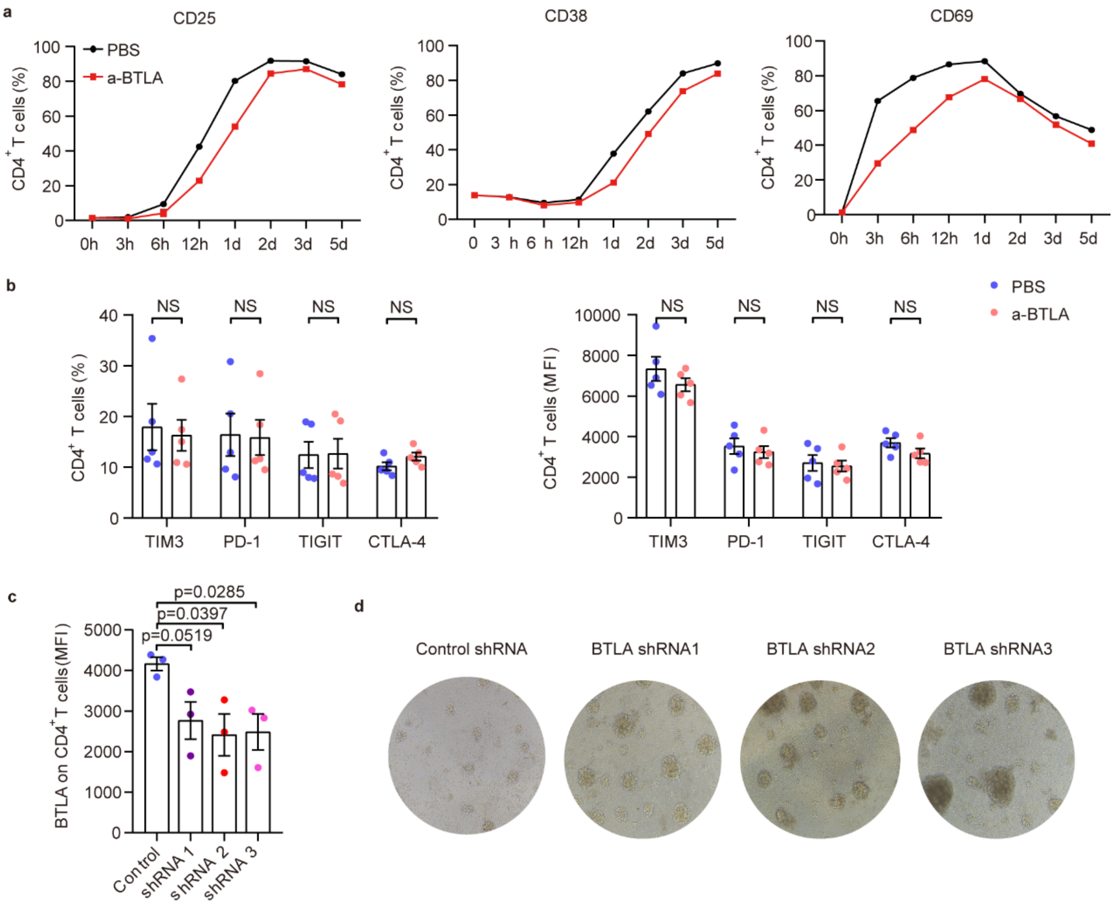

Supplementary Fig. 7 Time- and dose-dependent inhibition of CD4$^+$ T-cell activation by anti-BTLA. (a) Crosslinking of BTLA showed the strongest ability to suppress CD4$^+$ T-cell activation upon 1 day of anti-BTLA stimulation (a, *n* = 2 donors). (b) Crosslinking of BTLA did not result in changes in the expression of programmed cell death protein-1 (PD-1), cytotoxic T-lymphocyte antigen 4 (CTLA-4), T-cell immunoglobulin and mucin-domain-containing-3 (TIM-3), or T-cell immunoglobulin and ITIM domain (TIGIT) (*n* = 5 donors). (c) Three specific BTLA shRNAs could inhibit the expression of BTLA on CD4$^+$ T cells (*n* = 3 donors). (d) After crosslinking of BTLA using an agonistic anti-BTLA monoclonal antibody, the proliferation of CD4$^+$ T cells was significantly increased in the three specific BTLA shRNA groups compared with that in the control shRNA group. Data were calculated as mean ± SEM (b, c), Mann–Whitney *U* test (b, c). A two-sided *P* value < .05 was considered significant.

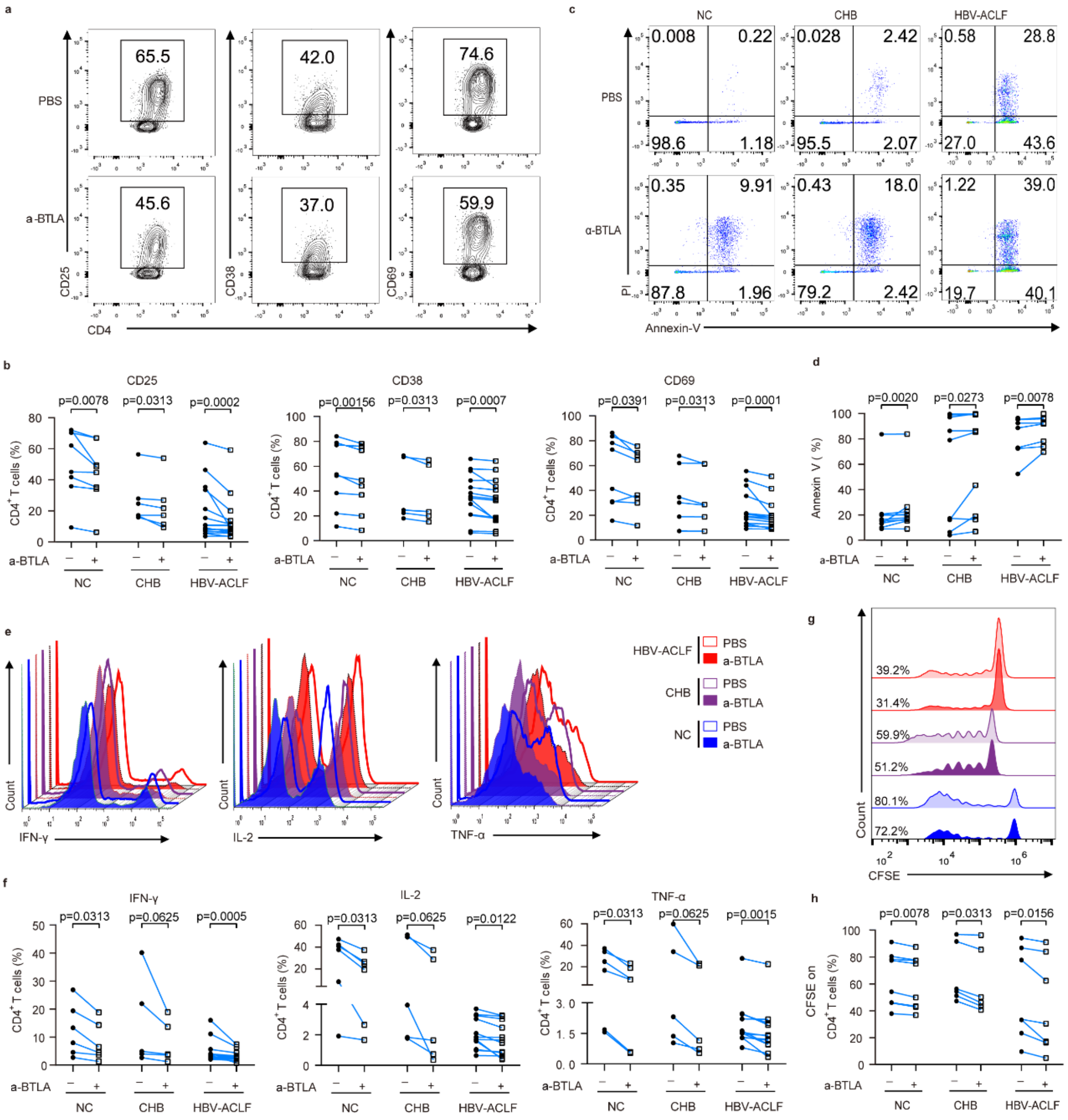

Supplementary Fig. 8 BTLA inhibited activation, proliferation, and secretory cytokines but promoted the apoptosis of CD4$^+$ T cells from the peripheral blood of NC, CHB, and HBV-ACLF patients. (a, b) Contour plots (top) and bar graphs (bottom) showing that crosslinked BTLA markedly inhibited the expression of activation markers (CD25, CD38, and CD69, NC: *n* = 8 donors, CHB: *n* = 6 donors, HBV-ACLF: *n* = 14 donors). (c, d) Contour plots (top) and bar graphs (bottom) showing that crosslinked BTLA markedly promoted the apoptosis of CD4$^+$ T cells (NC: *n* = 10 donors, CHB: *n* = 10 donors, HBV-ACLF: *n* = 8 donors). (e, f) Contour plots (top) and bar graphs (bottom) showing that crosslinked BTLA markedly inhibited the production of IFN-γ, IL-2, and TNF-α induced by PMA/ionomycin (NC: *n* =6 donors, CHB: *n* =5 donors, HBV-ACLF: *n* =12 donors). (g, h) Contour plots (top) and bar graphs (bottom) showing that crosslinked BTLA markedly inhibited the proliferation of CD4$^+$ T cells (NC: *n* = 8 donors, CHB: *n* = 6 donors, HBV-ACLF: *n* = 8 donors). Wilcoxon test (b, d, f, h). A two-sided *P* value < .05 was considered significant.

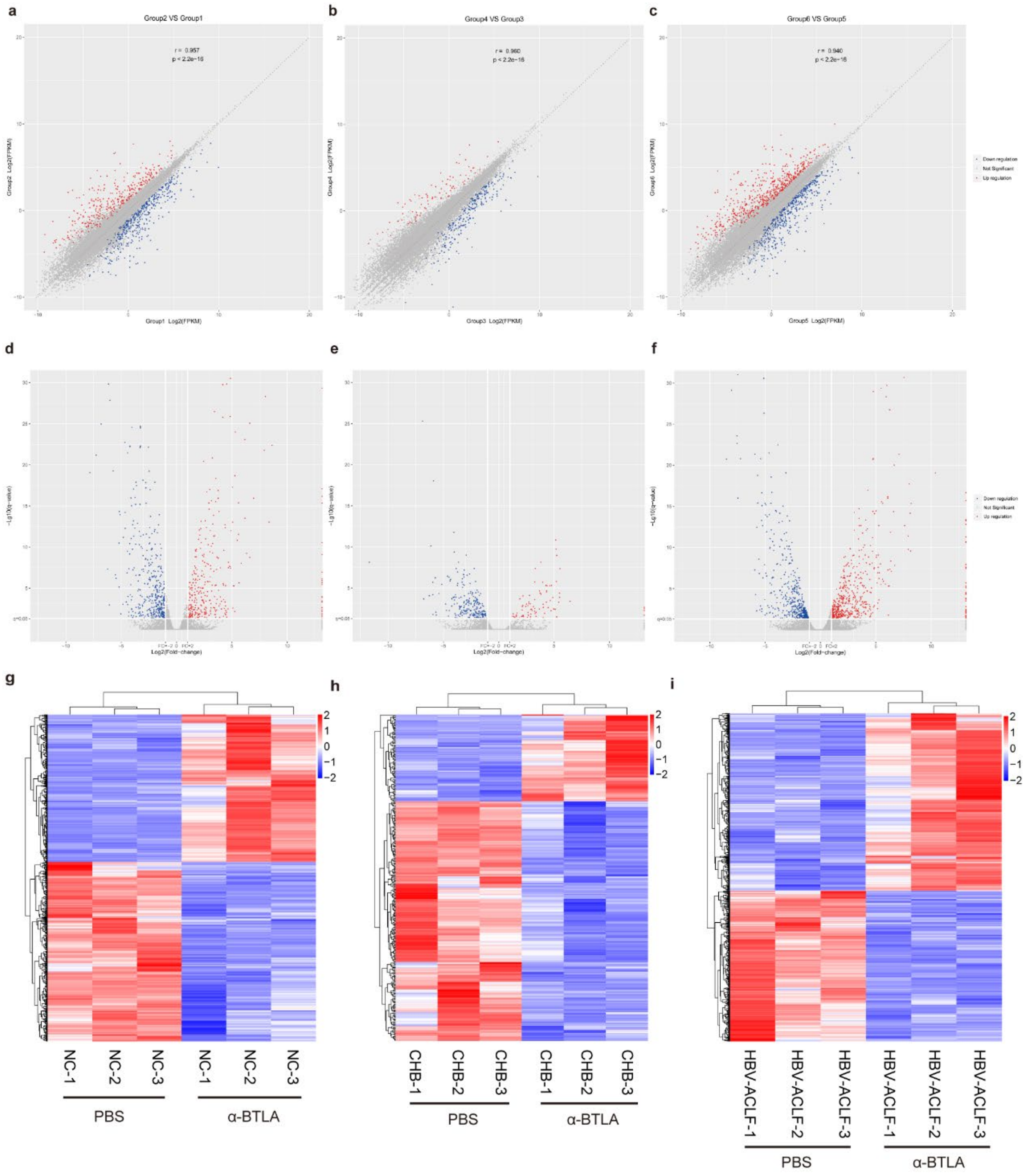

Supplementary Fig. 9 Anti-BTLA crosslinking increased gene expression changes in NC (*n* = 3 donors)**, CHB** (*n* = 3 donors)**, and HBV-ACLF patients** (*n* = 3 donors). Correlation, volcano plot, and heatmap of gene expression of PBMC with or without anti-BTLA crosslinking from NC (a, d, g), CHB (b, e, h), and HBV-ACLF patients (c, f, i) are shown.

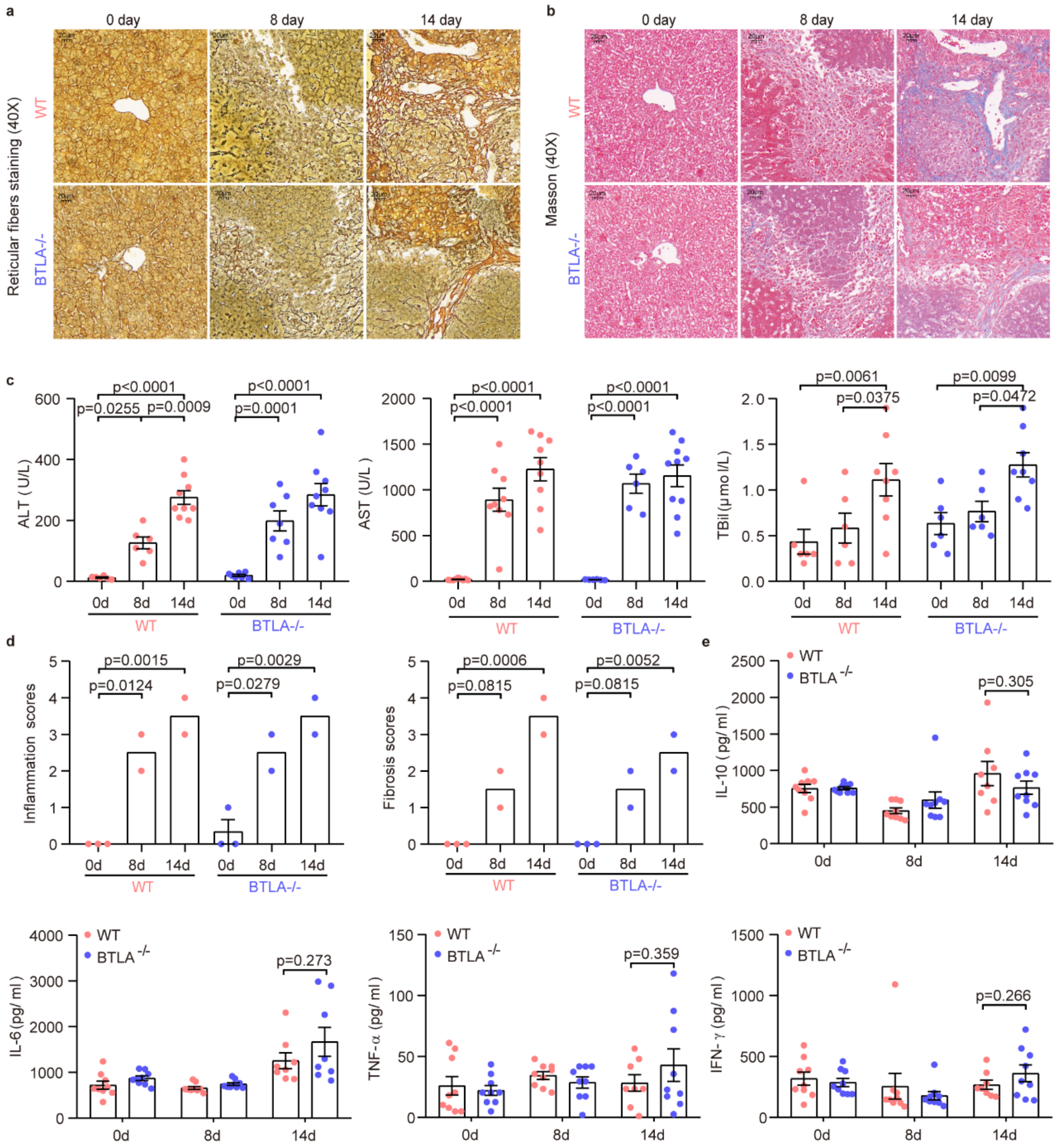

Supplementary Fig. 10 Characterization of a mouse model of ACLF induced by Concanavalin A (ConA). (a, b) Reticular fiber (left) and Masson staining (right) of liver pathology at baseline, 8 days, and 14 days in WT and BTLA$^{-/-}$ C57BL/6 mice. (c, d) Serum Alanine transaminase (ALT), Aspartate transaminase (AST), and TBil levels (WT: n = 9 mice, BTLA$^{-/-}$: n = 9 mice), inflammation, and fibrosis scores (WT: n = 3 mice, BTLA$^{-/-}$: n = 3 mice) were measured at baseline, 8 and 14 days post-ConA injection. (e) Cytokine (TNF-α, IL-6, and IFN-γ) levels were slightly increased, while IL-10 levels were slightly decreased in the plasma of BTLA$^{-/-}$ mice (n = 10 mice) compared to those in WT mice (n = 10 mice) at day 14. Data were calculated as mean ± SEM (c, d, e), Two-way ANOVA followed by Sidak's multiple-comparison test (c, d), Mann–Whitney *U* test (e). A two-sided *P* value < .05 was considered significant.

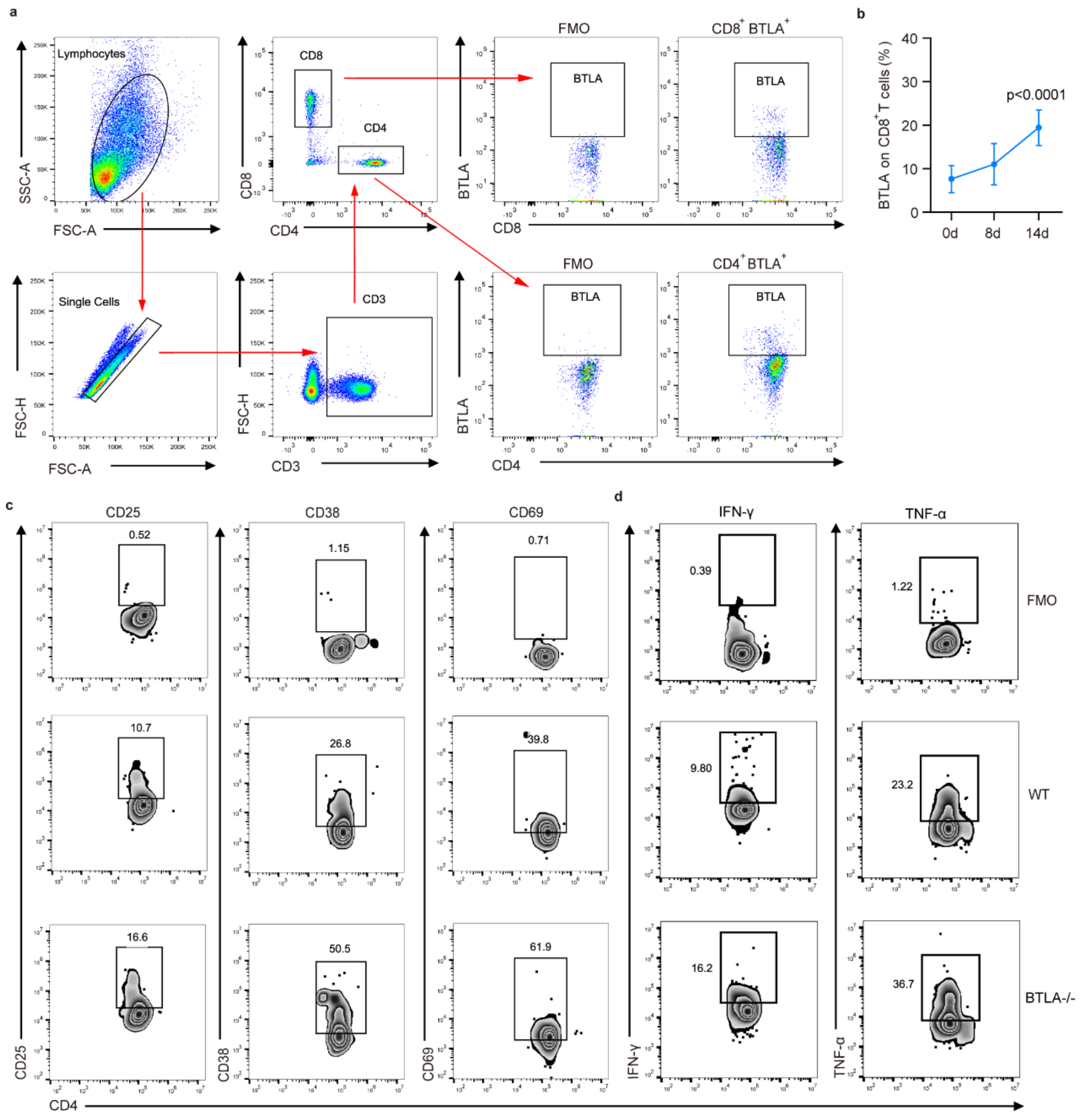

Supplementary Fig. 11 BTLA expression significantly increased on circulating CD4$^+$/CD8$^+$ T cells in ACLF model induced by ConA. (a) Flow cytometry diagram of BTLA-expressing CD4$^+$/CD8$^+$ T cells from peripheral blood of WT mice. (b) Expression of BTLA on CD8$^+$ T cells was significantly increased on days 14 compared to the baseline in WT mice (n = 9 mice) following ConA injection. (c, d) Contour plots showing that the percentages of activation indices (CD25, CD38, and CD69) and cytokines (IFN-γ and TNF-α) were higher in BTLA$^{-/-}$ mice than in WT mice following ConA injection on day 14. Data were calculated as mean ± SEM (b), Kruskal-Wallis H test followed by Dunn's multiple comparison test (b). A two-sided *P* value < .05 was considered significant.

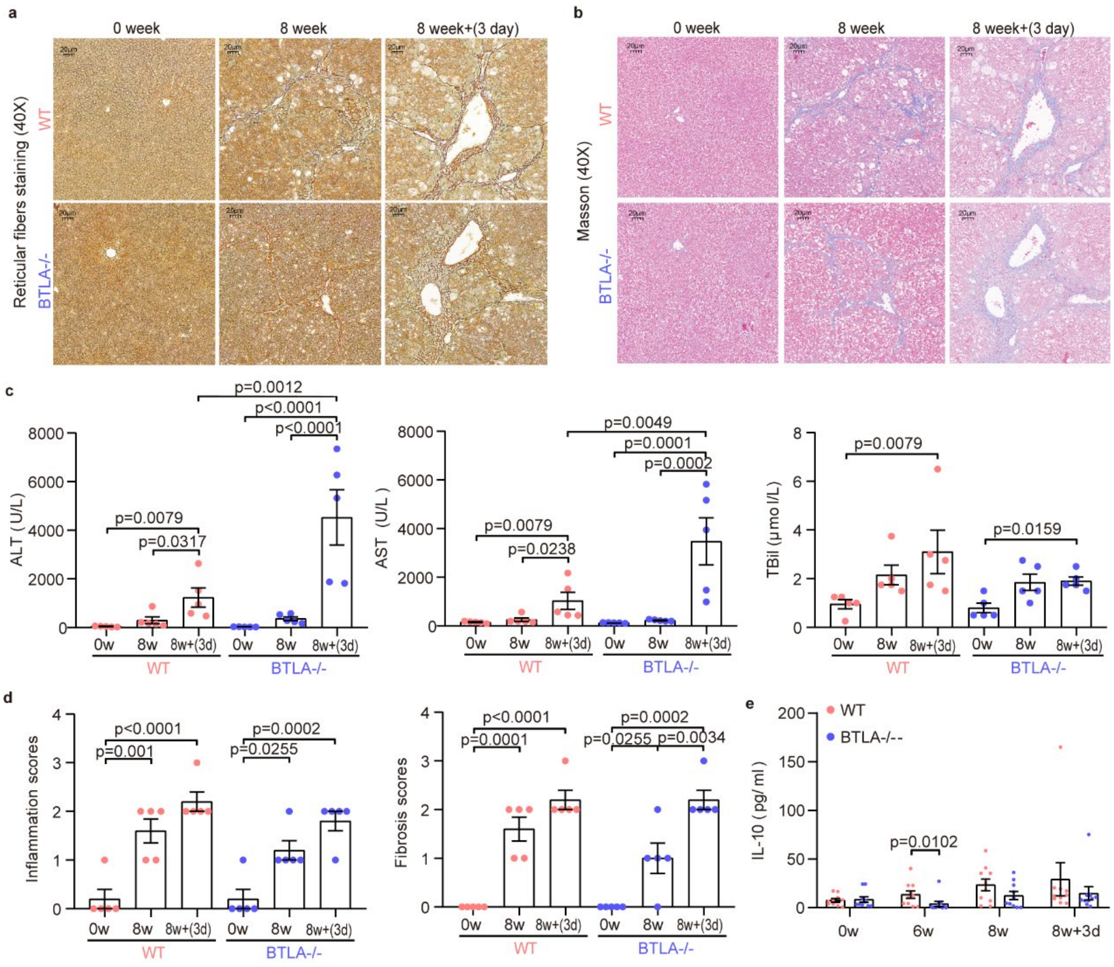

Supplementary Fig. 12 Characterization of a mouse model of ACLF induced by carbon tetrachloride (CCl_4). (a, b) Reticular fiber (left) and Masson staining (right) of liver pathology at baseline, 8 weeks, and 8 weeks + 3 days in WT and BTLA$^{-/-}$ C57BL/6 mice. (c, d) Serum ALT, AST, and TBil levels, inflammation, and fibrosis scores were measured at baseline, 8 weeks, and 8 weeks + 3 days (WT: *n* = 5 mice, BTLA$^{-/-}$: *n* = 5 mice). (e) Level of IL-10 was measured at baseline, 8 weeks, and 8 weeks + 3 days (WT: *n* = 10 mice, BTLA$^{-/-}$: *n* = 10 mice). Kruskal-Wallis H test followed by Dunn's multiple comparison test (c, d), and Mann–Whitney *U* test (e). A two-sided *P* value < .05 was considered significant.

Supplementary Table 1. Subject demographics and clinical characteristics

Group	NC (*n* = 90)	CHB (*n* = 104)	HBV-ACLF (*n* = 71)
Sex (Male, %)	29 (32.22%)	82 (78.85%) [c]	58 (81.69%) [b]
Age (years)	30.00 (26.0–43.5)	31.00 (26.00–40.75)	45.00 (35.00–52.25) [a, b]
Hepatitis B virus s antigen (IU/mL）	-	7250.00 (2240.38–20801.67)	1141.76 (250.00–6055.56)
Hepatitis B virus e antigen (positive, %)	-	81 (77.88%)	28 (39.44%) [a]
HBV DNA (Lg IU/mL)	-	7.34 (4.59–8.21)	3.69 (2.70–6.13) [a]
Albumin (g/L)	-	41.50 (39.30–44.50)	33.00 (30.55–38.73) [a]
Total bilirubin (μmol/L)	-	18.80 (12.70–40.95)	365.70 (279.70–462.40) [a]
Alanine aminotransferase (IU/L)	-	229.00 (126.50–473.50)	103.00 (54.50–414.50) [a]
Aspartate aminotransferase (IU/L)	-	110.00 (51.00–217.50)	107.50 (65.75–231.00)
Alkaline phosphatase (U/L)	-	90.00 (71.00–122.00)	131.00 (104.50–156.00) [a]
γ-Glutamyl transferase (U/L)	-	72.00 (23.00–160.00)	69.00 (44.50–97.00)
Creatinine (μmol/L)	-	81.70 (70.00–91.10)	73.20 (59.55–93.80)
Cholinesterase (U/L)	-	6454.50 (5547.50–7372.00)	2895.00 (2155.00–3745.00) [a]
White blood cell count (10^9/L)	-	5.60 (4.50–6.70)	6.09 (4.95–7.98) [a]
Neutrophil count (10^9/L)	-	2.82 (2.18–4.24)	4.03 (2.81–5.45) [a]
Hemoglobin (g/L)	-	147.00 (133.25–156.75)	122.50 (101.00–137.50) [a]
Platelet count (10^9/L)	-	179.50 (144.25–216.75)	84.00 (47.50–137.00) [a]
Prothrombin time (s)	-	11.40 (10.90–12.20)	22.50 (19.40–27.90) [a]
International normalized ratio	-	1.03 (0.98–1.10)	2.09 (1.67–2.47) [a]
C-reactive protein (mg/L)	-	3.58 (2.27–5.53)	11.05 (6.50–16.93) [a]
Procalcitonin (ng/mL)	-	-	0.63 (0.45–0.97)
Good prognosis n (%)	-	-	32 (45.07%)
Bacterial infection n (%)	-	-	44 (61.97%)
Ascites n (%)	-	-	44 (61.97%)
Portal hypertension n (%)	-	-	47 (66.20%)
Hepatic encephalopathy n (%)	-	-	12 (16.90%)
ACLF grade 1/2/3 (n)	-	-	48/15/8
Child-pugh score	-	-	11.0 (10.0–12.0)
MELD score	-	-	23.94 (20.61–28.50)
CLIF-SOFA	-	-	10.00 (7.00–11.00)
CLIF-C ACLFs	-	-	36.53 (32.64–44.95)
COSSH-ACLFs	-	-	8.69 (7.12–9.88)

Results are expressed as medians and interquartile ranges. [a] Significant differences when HBV-ACLF patients were compared to CHB patients; [b] Significant differences when HBV-ACLF patients were compared to NC.

Supplementary Table 2. Detailed information about all antibodies

Antibodies	Catalogue numbers	Clone numbers	Suppliers	Dilutions
APC anti-human CD3	317318	OKT3	Biolegend	1:100
BV510™ anti-human CD4	562970	SK3	BD Biosciences	1:100
PE/Cy7 anti-human CD8	566858	HIT8a	BD Biosciences	1:100
Percp/Cy5.5 anti-human BTLA	344514	MIH26	Biolegend	1:100
FITC anti-human CD27	302806	O323	Biolegend	1:100
APC/Cy7 anti-human CD45RA	304128	HI100	Biolegend	1:100
APC/FireTM 750 anti-human CD45	982314	HI30	Biolegend	1:100
BV510™ anti-human CCR4	359416	L291H4	Biolegend	1:100
APC/Cy7 anti-human CCR6	353432	G034E3	Biolegend	1:100
PE anti-human CCR10	341504	6588-5	Biolegend	1:100
BV421™ anti-human CXCR3	353716	G025H7	Biolegend	1:100
PE/Cy7 anti-human CXCR5	356924	J252D4	Biolegend	1:100
APC AF750 anti-human CD3	A66329	UCHT1	Beckman	1:100
ECD anti-human CD4	6604727	SFCI12T4D11	Beckman	1:100
FITC anti-human CCR5	359120	J418F1	Biolegend	1:100
PE anti-human BTLA	344506	MIH26	Biolegend	1:100
PC5 anti-human CD127	A64617	R34.34	Beckman	1:100
PC7 anti-human CD64	B06025	22	Beckman	1:100
APC anti-human CD25	B09684	B09684	Beckman	1:100
APC A700 anti-human CD7	A70201	8H8.1	Beckman	1:100
PB anti-human CD57	A74779	NC1	Beckman	1:100
FITC anti-human CD3	300406	UCHT1	Biolegend	1:100
PerCP anti-human CD4	300527	RPA-T4	Biolegend	1:100
APC/Cyanine7 anti-human CD8a	300925	HIT8a	Biolegend	1:100
APC anti-human CD270 (HVEM)	318807	122	Biolegend	1:100
PE/Cyanine7 anti-human CD86	305421	IT2.2	Biolegend	1:100
Brilliant Violet 421™ anti-human CD80	305221	2D10	Biolegend	1:100
PE anti-human CD56	985902	QA17A16	Biolegend	1:100
CFSE	C34554		Thermo	1:100
BV421 anti-human IFN-γ	562988	B27	BD Biosciences	1:100
APC/Cy7 anti-human TNF-α	502944	MAb11	BD Biosciences	1:100
PE anti-human IL-2	560902	MQ1-17H12	BD Biosciences	1:100
PE anti-human CD25	557138	M-A251	BD Biosciences	1:100
BV421 anti-human CD38	562444	HIT2	BD Biosciences	1:100
APC/Cy7 anti-human CD69	557756	FN50	BD Biosciences	1:100
FITC anti-human Annexin V	556547	RUO	BD Biosciences	1:100
PE anti-human PI	556547	RUO	BD Biosciences	1:100
FITC anti-human CD272 (BTLA) Antibody	344523	MIH26	Biolegend	1:100
APC anti-Human CD279 (PD-1)	70-F11279A03-25	J110	MultiSciences	1:100

PE anti-Human CD152 (CTLA-4)	70-F1115202-25	BNI3	MultiSciences	1:100
Brilliant Violet 421 anti-human TIGIT (VSTM3)	372709	A15153G	Biolegend	1:100
PE-Cyanine7 anti-human CD366 (TIM3)	25-3109-41	F38-2E2	eBioscience	1:100
PerCP-cy5.5 anti-Human CD4	70-F11004A04/2-25	SK3	MultiSciences	1:100
PerCP/Cyanine5.5 anti-mouse CD3ε	20201221	145-2C11	Biolegend	1:100
FITC anti-mouse CD4	20201221	RM4-5	Biolegend	1:100
APC/Cyanine7 anti-mouse CD8a	20201221	53-6.7	Biolegend	1:100
PE anti-mouse IFN-γ	20201221	XMG1.2	Biolegend	1:100
Brilliant Violet 421™ anti-mouse TNF-α	20201221	MP6-XT22	Biolegend	1:100
PE/Cyanine7 anti-mouse IL-2	20201221	JES6-5H4	Biolegend	1:100
PE/Cyanine7 anti-mouse CD25	20201221	PC61	Biolegend	1:100
Brilliant Violet 421™ anti-mouse CD38	20201221	90	Biolegend	1:100
PE anti-mouse CD69	20201221	H1.2F3	Biolegend	1:100
anti-BTLA antibodies	ab212089	EPR20539	Abcam	1:1000
PI3K	4257S	-	Cell Signaling Technology	1:1000
phospho-PI3K	13857S	-	Cell Signaling Technology	1:1000
Akt	4691s	-	Cell Signaling Technology	1:1000
phospho-Akt	4060S	-	Cell Signaling Technology	1:2000
phospho-GSK-3β	9336S	-	Cell Signaling Technology	1:1000
CREB	9197S	-	Cell Signaling Technology	1:1000
phospho-CREB	9198S	-	Cell Signaling Technology	1:1000
phospho-SHP1	8849S	-	Cell Signaling Technology	1:1000
phospho-SHP2	5431T	-	Cell Signaling Technology	1:1000
GAPDH	9001-50-7	-	Biodesign International	1:1000

Supplementary Table 3. Specific primers for BTLA, stat3, 16S, and β-actin

Gene	Forward (5′-3′)	Reverse (5′-3′)
BTLA	TCTTTATGTGACAGGAAAGCAAA	CAGACCCTTCCTGCATCCTG
Stat3	CTTTGAGACCGAGGTGTATCACC	GGTCAGCATGTTGTACCACAGG
16S	AACTGGAGGAAGGTGGGGAT	AGGAGGTGATCCAACCGCA
β-actin	AGAGCTACGAGCT GCCTGAC	AGCACTGTGTTGGCGTACAG

References

1. Mahnke YD, Beddall MH, Roederer M. OMIP-017: human CD4(+) helper T-cell subsets including follicular helper cells. *Cytometry A* **83**, 439-440 (2013).
