# Supplementary material for: BTLA contributes to acute-on-chronic liver failure infection and mortality through CD4+ T-cell exhaustion
Source: Nat Commun. 2024 Feb 28;15:1835. doi: 10.1038/s41467-024-46047-8 (PMC10901893; doi:10.1038/s41467-024-46047-8)
Supplement: Supplementary file 3 — Reporting Summary [file 41467_2024_46047_MOESM3_ESM.pdf]

nature portfolio

Corresponding author(s): Jiming Zhang

Last updated by author(s): Nov 12, 2023

Reporting Summary

Nature Portfolio wishes to improve the reproducibility of the work that we publish. This form provides structure for consistency and transparency in reporting. For further information on Nature Portfolio policies, see our Editorial Policies and the Editorial Policy Checklist.

Statistics

For all statistical analyses, confirm that the following items are present in the figure legend, table legend, main text, or Methods section.

n/a	Confirmed	
☐	☒	The exact sample size (*n*) for each experimental group/condition, given as a discrete number and unit of measurement
☐	☒	A statement on whether measurements were taken from distinct samples or whether the same sample was measured repeatedly
☐	☒	The statistical test(s) used AND whether they are one- or two-sided<br>*Only common tests should be described solely by name; describe more complex techniques in the Methods section.*
☐	☒	A description of all covariates tested
☐	☒	A description of any assumptions or corrections, such as tests of normality and adjustment for multiple comparisons
☐	☒	A full description of the statistical parameters including central tendency (e.g. means) or other basic estimates (e.g. regression coefficient) AND variation (e.g. standard deviation) or associated estimates of uncertainty (e.g. confidence intervals)
☐	☒	For null hypothesis testing, the test statistic (e.g. *F*, *t*, *r*) with confidence intervals, effect sizes, degrees of freedom and *P* value noted<br>*Give P values as exact values whenever suitable.*
☒	☐	For Bayesian analysis, information on the choice of priors and Markov chain Monte Carlo settings
☐	☒	For hierarchical and complex designs, identification of the appropriate level for tests and full reporting of outcomes
☐	☒	Estimates of effect sizes (e.g. Cohen's *d*, Pearson's *r*), indicating how they were calculated

Our web collection on statistics for biologists contains articles on many of the points above.

Software and code

Policy information about availability of computer code

Data collection	GraphPad Prism version 7.0
Data analysis	GraphPad Prism version 7.0

For manuscripts utilizing custom algorithms or software that are central to the research but not yet described in published literature, software must be made available to editors and reviewers. We strongly encourage code deposition in a community repository (e.g. GitHub). See the Nature Portfolio guidelines for submitting code & software for further information.

Data

Policy information about availability of data

All manuscripts must include a data availability statement. This statement should provide the following information, where applicable:

- Accession codes, unique identifiers, or web links for publicly available datasets
- A description of any restrictions on data availability
- For clinical datasets or third party data, please ensure that the statement adheres to our policy

Data are available in the main text or the supplementary materials. Source data are provided with this paper.

April 2023

Research involving human participants, their data, or biological material

Policy information about studies with human participants or human data. See also policy information about sex, gender (identity/presentation), and sexual orientation and race, ethnicity and racism.

Reporting on sex and gender	In human research, we only use sex to describe the composition ratio of males (sex, male, %) in Table 1.
Reporting on race, ethnicity, or other socially relevant groupings	No related report.
Population characteristics	There were more males in the group of HBV-ACLF patients than in the NC groups, while there was no significant difference in the proportions of males between CHB and HBV-ACLF patients. HBV-ACLF patients were older than the CHB and HC groups. Moreover, compared with CHB patients, HBV-ACLF patients showed significantly higher physiological and biochemical indicators of liver injury (TBil, DBil, ALT, AST, PT, and INR) but markedly lower compensatory indices of liver function (ALB, Hgb, and CHE) and virological parameters (HBsAg, HBeAg, and HBV DNA). Circulating inflammatory markers (WBC, neutrophil count and CRP) were significantly increased in HBV-ACLF patients compared with CHB patients.
Recruitment	We included 101 patients with severe liver injury (TBil ≥ 5 mg/dL and INR ≥ 1.5), including 71 patients with HBV-ACLF meeting COSSH criteria (TBil≥ 12 mg/dL and INR ≥ 1.5, regardless of the presence of cirrhosis, complicated by hepatic and/or extrahepatic organ failure). Because of the differences between Chinese and western races, the diagnostic criteria of HBV-ACLF are different. So three diagnostic criteria were developed: COSSH standard, North American Consortium for the Study of End-stage Liver Disease (NACSELD) Criteria and the Chronic Liver Failure-sequential Organ Failure Assessment (CLIF-SOFA) criteria.
Ethics oversight	The study protocol was approved by the Ethics Committee of Huashan Hospital, affiliated with Fudan University (KY2021-652) and Biomedical Research Affiliated to Fujian Medical University (No.2014-87). Written consent was obtained from healthy individuals and patients with CHB and HBV-ACLF.

Note that full information on the approval of the study protocol must also be provided in the manuscript.

Field-specific reporting

Please select the one below that is the best fit for your research. If you are not sure, read the appropriate sections before making your selection.

☒ Life sciences ☐ Behavioural & social sciences ☐ Ecological, evolutionary & environmental sciences

For a reference copy of the document with all sections, see nature.com/documents/nr-reporting-summary-flat.pdf

Life sciences study design

All studies must disclose on these points even when the disclosure is negative.

Sample size	Three groups of research objects, according to statistical calculations, generally more than 30 research objects in each group is statistically significant, so ninety healthy individuals without any apparent disease, 104 patients with CHB and 101 with severe liver injury TBil ≥ 5 mg/dL and INR ≥ 1.5) were enrolled this study.
Data exclusions	Patients were excluded if they were HBV coinfected with any other virus (e.g., Hepatitis A, C, and D virus), had drug or alcohol-induced liver diseases, had hepatocellular carcinoma or other tumors, or were treated with immunosuppressive drugs
Replication	We used three diagnostic criteria for ACLF to confirm the high expression of BTLA in ACLF patients. At the same time, we used two different methods to construct ACLF animal models, and both confirmed that WT knockout was not easy to form immune exhaustion and secondary infection.
Randomization	We select patients based on inclusion criteria, and all of these subjects were enrolled at the same time (from January 2014 to September 2018), so there is no randomization involved.
Blinding	We select patients based on inclusion criteria, and this study was not a controlled clinical study to evaluate the merits of two or more drugs, so it did not involve blinding

Reporting for specific materials, systems and methods

We require information from authors about some types of materials, experimental systems and methods used in many studies. Here, indicate whether each material, system or method listed is relevant to your study. If you are not sure if a list item applies to your research, read the appropriate section before selecting a response.

Materials & experimental systems

n/a	Involved in the study	
☐	☒	Antibodies
☒	☐	Eukaryotic cell lines
☒	☐	Palaeontology and archaeology
☐	☒	Animals and other organisms
☐	☒	Clinical data
☒	☐	Dual use research of concern
☒	☐	Plants

Methods

n/a	Involved in the study	
☒	☐	ChIP-seq
☐	☒	Flow cytometry
☒	☐	MRI-based neuroimaging

Antibodies

Antibodies used

APC anti-human CD3, BV510™ anti-human CD4, PE/Cy7 anti-human CD8, Percp/Cy5.5 anti-human BTLA, FITC anti-human CD27, APC/Cy7 anti-human CD45RA, APC/FireTM 750 anti-human CD45, BV510™ anti-human CCR4, APC/Cy7 anti-human CCR6, PE anti-human CCR10, BV421™ anti-human CXCR3, PE/Cy7 anti-human CXCR5, APC AF750 anti-human CD3, ECD anti-human CD4, FITC anti-human CCR5, PE anti-human BTLA, PC5 anti-human CD127, PC7 anti-human CD64, APC anti-human CD25, APC A700 anti-human CD7, PB anti-human CD57, FITC anti-human CD3, PerCP anti-human CD4, APC/Cyanine7 anti-human CD8a, APC anti-human CD270 (HVEM, TR2), PE/Cyanine7 anti-human CD86, Brilliant Violet 421™ anti-human CD80, PE anti-human CD56, CFSE, BV421 anti-human IFN-γ APC/Cy7 anti-human TNF-α, PE anti-human IL-2, PE anti-human CD25, BV421 anti-human CD38, APC/Cy7 anti-human CD69, FITC anti-human Annexin V , PE anti-human PI, FITC anti-human CD272 (BTLA) Antibody, APC anti-Human CD279 (PD-1) , PE anti-Human CD152 (CTLA-4) , Brilliant Violet 421 anti-human TIGIT (VSTM3), PE-Cyanine7 anti-human CD366 (TIM3), PerCP-cy5.5 anti-Human CD4
PerCP/Cyanine5.5 anti-mouse CD3ε, FITC anti-mouse CD4, APC/Cyanine7 anti-mouse CD8a, PE anti-mouse IFN-γ, Brilliant Violet 421™ anti-mouse TNF-α, PE/Cyanine7 anti-mouse IL-2, PE/Cyanine7 anti-mouse CD25, Brilliant Violet 421™ anti-mouse CD38, PE anti-mouse CD69, anti-BTLA antibodies, PI3K, phospho-PI3K, Akt, phospho-Akt, phospho-GSK-3β, CREB, phospho-CREB, phospho-SHP1 phospho-SHP2, GAPDH

Validation

antibodies,catalogue numbers,Clone numbers,suppliers,dilutions
APC anti-human CD3, 317318, OKT3, Biolegend,1:100
BV510™ anti-human CD4, 562970, SK3, BD Biosciences,1:100
PE/Cy7 anti-human CD8, 566858, HIT8a, BD Biosciences,1:100
Percp/Cy5.5 anti-human BTLA, 344514, MIH26, Biolegend,1:100
FITC anti-human CD27, 302806, O323, Biolegend,1:100
APC/Cy7 anti-human CD45RA, 304128, HI100, Biolegend,1:100
APC/FireTM 750 anti-human CD45, 982314, HI30, Biolegend, 1:100
BV510™ anti-human CCR4 359416, L291H4, Biolegend,1:100
APC/Cy7 anti-human CCR6 353432, G034E3, Biolegend,1:100
PE anti-human CCR10 341504, 6588-5, Biolegend,1:100
BV421™ anti-human CXCR3, 353716, G025H7, Biolegend,1:100
PE/Cy7 anti-human CXCR5, 356924, J252D4, Biolegend,1:100
APC AF750 anti-human CD3, A66329, UCHT1, Beckman,1:100
ECD anti-human CD4, 6604727, SFCI12T4D11, Beckman,1:100
FITC anti-human CCR5, 359120, J418F1, Biolegend,1:100
PE anti-human BTLA, 344506, MIH26, Biolegend,1:100
PC5 anti-human CD127, A64617, R34.34, Beckman,1:100
PC7 anti-human CD64, B06025, 22, Beckman,1:100
APC anti-human CD25, B09684, B09684, Beckman,1:100
APC A700 anti-human CD7, A70201, 8H8.1, Beckman,1:100
PB anti-human CD57, A74779, NC1, Beckman,1:100
FITC anti-human CD3, 300406, UCHT1, Biolegend,1:100
PerCP anti-human CD4, 300527, RPA-T4, Biolegend,1:100
APC/Cyanine7 anti-human CD8a, 300925, HIT8a, Biolegend,1:100
APC anti-human CD270 (HVEM, TR2), 318807, 122, Biolegend,1:100
PE/Cyanine7 anti-human CD86, 305421, IT2.2, Biolegend,1:100
Brilliant Violet 421™ anti-human CD80, 305221, 2D10, Biolegend,1:100
PE anti-human CD56, 985902, QA17A16, Biolegend,1:100
CFSE, C34554, Thermo,1:100
BV421 anti-human IFN-γ, 562988, B27, BD Biosciences,1:100
APC/Cy7 anti-human TNF-α, 502944, MAb11, BD Biosciences,1:100
PE anti-human IL-2, 560902, MQ1-17H12, BD Biosciences1:100
PE anti-human CD25, 557138, M-A251, BD Biosciences,1:100
BV421 anti-human CD38, 562444, HIT2, BD Biosciences,1:100
APC/Cy7 anti-human CD69, 557756, FN50, BD Biosciences,1:100
FITC anti-human Annexin V, 556547, RUO, BD Biosciences,1:100
PE anti-human PI, 556547, RUO, BD Biosciences,1:100
FITC anti-human CD272 (BTLA), 344523, MIH26, Biolegend,1:100

APC anti-Human CD279 (PD-1) , 70-F11279A03-25, J110, MultiSciences,1:100<br>PE anti-Human CD152 (CTLA-4), 70-F1115202-25, BNI3, MultiSciences,1:100<br>Brilliant Violet 421 anti-human TIGIT (VSTM3), 372709, A15153G, Biolegend,1:100<br>PE-Cyanine7 anti-human CD366 (TIM3) , 25-3109-41, F38-2E2, eBioscience,1:100<br>PerCP-cy5.5 anti-Human CD4, 70-F11004A04/2-25, SK3, MultiSciences,1:100<br>PerCP/Cyanine5.5 anti-mouse CD3ε, 20201221, 145-2C11, Biolegend,1:100<br>FITC anti-mouse CD4, 20201221, RM4-5, Biolegend,1:100<br>APC/Cyanine7 anti-mouse CD8a, 20201221, 53-6.7, Biolegend,1:100<br>PE anti-mouse IFN-γ, 20201221, XMG1.2, Biolegend,1:100<br>Brilliant Violet 421™ anti-mouse TNF-α, 20201221, MP6-XT22, Biolegend,1:100<br>PE/Cyanine7 anti-mouse IL-2, 20201221, JES6-5H4, Biolegend,1:100<br>PE/Cyanine7 anti-mouse CD25, 20201221, PC61, Biolegend,1:100<br>Brilliant Violet 421™ anti-mouse CD38, 20201221, 90, Biolegend,1:100<br>PE anti-mouse CD69, 20201221, H1.2F3, Biolegend,1:100<br>anti-BTLA antibodies, ab212089, EPR20539, Abcam, USA, 1:1000<br>PI3K, 4257S, Cell Signaling Technology, Beverly, Mass, 1:1000<br>phospho-PI3K, 13857S, Cell Signaling Technology, Beverly, Mass, 1:1000<br>Akt 4691s Cell Signaling Technology, Beverly, Mass 1:1000<br>phospho-Akt 4060S   Cell Signaling Technology, Beverly, Mass 1:2000<br>phospho-GSK-3β 9336S Cell Signaling Technology, Beverly, Mass 1:1000<br>CREB 9197S Cell Signaling Technology, Beverly, Mass 1:1000<br>phospho-CREB 9198S Cell Signaling Technology, Beverly, Mass 1:1000<br>phospho-SHP1 8849S Cell Signaling Technology, Beverly, Mass 1:1000<br>phospho-SHP2 5431T Cell Signaling Technology, Beverly, Mass 1:1000<br>GAPDH 9001-50-7 Biodesign International, Saco, Maine 1:1000

Animals and other research organisms

Policy information about studies involving animals; ARRIVE guidelines recommended for reporting animal research, and Sex and Gender in Research

Laboratory animals	Male wild-type (WT) and BTLA-/- C57BL/6 mice, both 8 weeks old and weighing 20 ± 1 g, were purchased from the Shanghai Model Organisms Center, Inc (Shanghai, China).
Wild animals	temperature: 20-26 ℃, humidity: 40-70, dark/photoperiod: 12h/12h
Reporting on sex	we chose male mice to do the experiment.
Field-collected samples	They were bred in a specific pathogen-free barrier facility.
Ethics oversight	All animal experiments were approved by the Ethics Committee of Shanghai Public Health Clinical Center and the Institutes of Biomedical Science, Shanghai Medical College, Fudan University.

Note that full information on the approval of the study protocol must also be provided in the manuscript.

Clinical data

Policy information about clinical studies

All manuscripts should comply with the ICMJEguidelines for publication of clinical research and a completedCONSORT checklist must be included with all submissions.

Clinical trial registration	It is not a clinical trial.
Study protocol	Not available. We only compared the expression levels of BTLA on the immune cells of NC, CHB and HBV-ACLF groups
Data collection	Blood samples were collected and PBMC were isolated from 104 patients with CHB and 101 with severe liver injury TBil ≥ 5 mg/dL and INR ≥ 1.5) within 24 h of admission to the Department of Infectious Diseases, Huashan Hospital affiliated to Fudan University and First Hospital of Quanzhou Affiliated to Fujian Medical University from January 2014 to September 2018. Ninety healthy individuals without any apparent disease were enrolled as normal controls.
Outcomes	Expression of BTLA on CD4+ T cells and HVEM on dendritic cells (DC) is elevated synchronously in HBV-ACLF

Flow Cytometry

Plots

Confirm that:

- [ ] The axis labels state the marker and fluorochrome used (e.g. CD4-FITC).
- [x] The axis scales are clearly visible. Include numbers along axes only for bottom left plot of group (a 'group' is an analysis of identical markers).
- [x] All plots are contour plots with outliers or pseudocolor plots.
- [x] A numerical value for number of cells or percentage (with statistics) is provided.

Methodology

Sample preparation	Peripheral blood mononuclear cells (PBMC) were isolated from ethylenediamine tetraacetic acid-anticoagulant venous blood using Ficoll-Hypaque density gradient centrifugation (Cedarlane Laboratories). Fresh liver tissues were cut out. Next, the single-cell suspension was collected and washed with phosphate-buffered saline. Afterward, the cell suspension was resuspended in 40% Percoll (Sigma-Aldrich) and gently overlaid onto 70% Percoll and centrifuged for 20 min at 2000 rpm. Liver-infiltrating lymphocytes were collected from the interface.
Instrument	Peripheral blood CD4+T cells were purified using magnetic beads (Miltenyi Biotec);<br>Data acquisition and analysis were performed with MoFlo XDP (Beckman, USA);<br>Levels of pro-inflammatory cytokines were determined using the Luminex 200 multiplexing instrument (EMD Millipore, USA)<br>RNA sequencing (RNA-Seq) was performed on an Illumina Hiseq 2000 platform
Software	GraphPad Prism 7.0 (GraphPad Software Inc., San Diego, California, USA)
Cell population abundance	Peripheral blood CD4+T cells were purified using magnetic beads (Miltenyi Biotec) at a purity level of ≥90%
Gating strategy	We set up a cell collection strategy where the collection stops when all cells reach 50,000.

- [x] Tick this box to confirm that a figure exemplifying the gating strategy is provided in the Supplementary Information.
